# Chemical intolerance: involvement of brain function and networks after exposure to extrinsic stimuli perceived as hazardous

**DOI:** 10.1186/s12199-019-0816-6

**Published:** 2019-10-22

**Authors:** Kenichi Azuma, Iwao Uchiyama, Mari Tanigawa, Ikuko Bamba, Michiyo Azuma, Hirohisa Takano, Toshikazu Yoshikawa, Kou Sakabe

**Affiliations:** 1 Department of Environmental Medicine and Behavioral Science, Kindai University Faculty of Medicine, 377-2 Ohnohigashi, Osakasayama, Osaka 589-8511 Japan; 2 Sick-house Medical Science Laboratory, Division of Basic Research, Louis Pasteur Center for Medical Research, Kyoto, 606-8225 Japan; 3 Outpatient Department of Sick-house Syndrome, Hyakumanben Clinic, Kyoto, 606-8225 Japan; 4 Clinical Immune Function Laboratory, Division of Basic Research, Louis Pasteur Center for Medical Research, Kyoto, 606-8225 Japan; 5 Faculty of Education, Home Economics, Tokyo Gakugei University, Koganei, 184-8501 Japan; 6 Department of Human Environmental Design, Faculty of Health Science, Kio University, Kitakatsuragi-gun, 635-0832 Japan; 7 Department of Environmental Engineering, Graduate School of Engineering, Kyoto University, Kyoto, Japan; 8 Department of Anatomy and Cellular Biology, Tokai University School of Medicine, Isehara, 259-1193 Japan

**Keywords:** Brain imaging, Chemical intolerance, Exposure event, Limbic system, Multiple chemical sensitivity, Odor processing, Prefrontal cortex, Psychosomatic symptoms, Sensory disruption, Susceptibility

## Abstract

**Background:**

Chemical intolerance (CI) is a chronic condition characterized by recurring and severe symptoms triggered by exposure to low levels of odorous or pungent substances. The etiology of CI has been a controversial subject for a long time. The aim of this review is to summarize findings on the neurological processing of sensory information during and after exposure to low levels of odorous or pungent substances in individuals with CI, focusing on the brain function and networks.

**Methods:**

Scientific studies on CI published between 2000 and 2019 in academic peer-reviewed journals were systematically searched using medical and scientific literature databases. Only peer-reviewed articles reporting original research from experimental human studies directly associated with CI, and involving related neurological responses or brain imaging after exposure to odorous or pungent substances (i.e., in chemical provocation tests), were considered.

**Results:**

Forty-seven studies were found to be eligible for a full-text review. Twenty-three studies met the selection criteria and were included in this review. Evidence indicated that differences between subjects with CI and healthy controls were observed by brain imaging during and after exposure to odorous or pungent substances. Differences in brain imaging were also observed between initial exposure and after exposure to these substances. Neurological processing of sensory information after exposure to extrinsic stimuli in the limbic system and related cortices were altered in subjects with CI. A previous documentable exposure event was likely to be involved in this alteration.

**Conclusions:**

This review documents consistent evidence for the altered neurological processing of sensory information in individuals with CI. Further neurophysiological research exploring the processing of extrinsic stimuli and cognition of sensation through the limbic system and related cortices in CI, and the appearance of symptoms in individuals with CI, are required.

## Background

Intolerance to odorous or pungent substances, known as chemical intolerance (CI), is a widespread occupational and public health problem and has been frequently reported in industrialized countries [1–3]. CI is a chronic acquired disorder characterized by nonspecific and recurrent symptoms in multiple organ systems associated with exposure to low levels of odorous or pungent substances (e.g., fragranced consumer products, cleaning products, combustion products, petroleum products, perfumes, softeners, new furniture, new newspapers, environmental tobacco smoke, building materials, organic solvents, pesticides, and car exhaust) at concentrations usually tolerated by most of the population [2, 4–6].

The type and severity of symptoms reported in response to exposure are highly variable. A number of symptoms involve the central nervous system (CNS) (e.g., headaches, dizziness, fatigue, irritability, cognitive deficit, anxiety, dyspnea, and difficulty concentrating) and are often combined with nonspecific symptoms from other organ systems, including the skin, mucosa/respiratory tract, musculoskeletal system, cardiovascular system, and gastrointestinal tract [2, 5, 7–10]. Between 8 and 33% of people in various populations consider themselves unusually sensitive to odorous or pungent substances, with the variability in prevalence depending largely on a wide variety of definitions and severity [3, 8, 11–22].

Severe CI is often referred to as multiple chemical sensitivity (MCS) [1, 2, 4, 6, 23]. Some groups prefer the name idiopathic environmental intolerance (IEI) to avoid confusion in diagnosis and etiology associated with the terms CI and MCS [24–28], because these terms imply unsupported judgments on causation, do not refer to a clinically defined disease, and are not based on accepted theories of underlying mechanisms or validated clinical criteria for diagnosis [29].

Thus, although the prevalence of nonclinical occurrence in the population is relatively high, the mechanisms behind CI and its diagnosis and etiology remain controversial and understudied. Most definitions are almost entirely qualitative, depending on subjective reports of symptoms and environmental exposure from patients and clinicians [1, 9, 30, 31]. However, various theories suggest that alterations in chemical sensory transduction and neural processing, rather than toxic processes, serve as key mechanisms of CI. Related experimental studies of subjects with CI have recently been reported [1, 31–37] and increased after the MCS 1999 consensus, which proposed consensus criteria for the definition of MCS [30]. In this article, we review findings on the neurological processing of sensory information in exposure to low levels of odorous or pungent substances in individuals with CI. In particular, we look at brain function and network activity after exposure to these substances.

## Methods

An online literature search was conducted across major electronic databases, including PubMed and Google Scholar, between January 1, 2000, and July 8, 2019. PubMed was primarily used to identify potential articles that met the search criteria, and others were used as complementary databases. The following key words were used as search criteria: “chemical intolerance” OR “chemical sensitivity.” A total of 871 articles were retrieved. The retrieved articles were reviewed by a reviewer (KA) in two stages: screening of titles and abstracts, followed by a full-text review. Additional articles were identified based on prior knowledge (e.g., documents or reports of international or national organizations) and by manual screening of the bibliographies of retrieved articles. After a thorough review of titles and abstracts, 47 studies were found to be eligible for a full-text review. Of these, 23 met our selection criteria and were included in this review (Table 1). Specifically, only peer-reviewed articles reporting original research from experimental human studies directly associated with CI or MCS, including IEI due to chemical exposure and involving related neurological responses or brain imaging after exposure to odorous or pungent substances (i.e., chemical provocation tests) were considered. Studies focusing on electromagnetic fields and noise, and not chemical provocation, were excluded.

**Table 1:** Summary of experimental human studies associated with CI and related neurological responses or brain imaging in chemical provocation tests

Study, year with reference	Type of analysis	Subjects (CI or MCS/control)	Substances	Exposure time	Measurement	Findings
Alessandrini et al. 2016 [38]	PET with^18^FDG uptake	26/11	Saline, vanillin	9 min	After 24 min of exposure	Different subcortical olfactory processing and an increased responsiveness in the central nervous system and olfactory center
Andersson et al. 2009 [39]	EEG, EOG	21/17	CO_2_, amyl acetate (banana smelling), sound	200 ms repetition, 72 stimuli during 1.5 h	During task	Attention bias and enhanced sensitization, and alterations in central, cognitive responses to chemical exposure
Andersson et al. 2014 [40]	fMRI	25/26	CO_2_, isoamyl acetate (banana smelling, below irritation threshold)	20 repetitions of 30 s	During task	Not characterized by hyperresponsiveness in sensory areas and interpreted as a limbic hyperactivity and speculatively as an inability to inhibit salient external stimuli
Andersson et al. 2016 [23]	Autonomic recordings	18/18	*n*-Butanol (below irritation threshold)	42 min	During task	Altered autonomic responses (higher pulse rate and lower pulse rate variability) and chemosensory perception during chemical exposure
Andersson et al. 2017 [41]	fMRI	14 olfactory sensitizers, 20 intermediate, and 15 habituaters	CO_2_, isoamyl acetate (banana smelling, below irritation threshold)	20 repetitions of 30 s	During task	In reanalysis of Andersson et al. (2014) [40], greater reactions in regions relevant for pain and saliency detection, and olfactory projection areas (olfactory region of the orbitofrontal cortex)
Azuma et al. 2013 [32]	fNIRS	12/11	Odorants (mandarin orange, perfume, Japanese cypress, and menthol)	10 s	During exposure	Activation in the prefrontal cortex during exposure. Poorer autonomic perception and negative affectivity. Altered prefrontal information processing associated with odor processing and memory and cognition processes
Azuma et al. 2015 [33]	fNIRS	6/6	Odorants (mandarin orange, perfume, Japanese cypress, and menthol)	10 s	After exposure	Activation in the orbitofrontal cortex after exposure. Altered prefrontal information processing associated with odor processing and memory and cognition processes
Azuma et al. 2016 [34]	fNIRS	10/6	Odorants (sweet and fecal)	10 s	During and after exposure	Activation in the prefrontal cortex and orbitofrontal cortex. Altered prefrontal information processing associated with odor processing and memory and cognition processes
Bornschein et al. 2008 [42]	Serum cortisol, cognitive performance	20/17	Solvent mixture of hydrocarbons (below odor threshold)	3 repetitions of 15 min	Before and after the exposure	No differences
Chiaravalloti et al. 2015 [43]	PET with^18^FDG uptake	26/11	Saline, vanillin	9 min	After 24 min of exposure	Different cortical olfactory processing with deactivation that mainly involves the frontal cortex and by active recruitment of the left inferior temporal gyrus
Claeson et al. 2017 [44]	SCA, sensory irritation	18/19	Acrolein, heptan	60 min	Before exposure, after and 24 h postexposure	No differences in SCA, greater sensory irritation, suggesting altered trigeminal reactivity
Claeson et al. 2017 [45]	Serum oxylipins and endocannabinoids	18/19	Acrolein, heptan	60 min	Before exposure, after and 24 h postexposure	No differences
Dantoft et al. 2015 [46]	Cytokine and chemokine in epithelial lining fluid	18/18	n-Butanol (below irritation threshold)	42 min	After 15 min of exposure	No abnormal upper airway inflammatory mediator levels
Dantoft et al. 2017 [47]	Gene expression for inflammatory markers	18/18	n-Butanol (below irritation threshold)	42 min	After 15 min of exposure	No differences in gene expression levels before/after exposure
Georgellis et al. 2003 [48]	Serum prolactin and cortisol	14/15	Furfuryl mercaptan, acetone, VOC mixture	20 min	Before and after exposure	No differences
Haumann et al. 2003 [49]	RR, HR	12/12	Ethyl benzene, 2-butanone, 2-propanol, 1-octanol (above odor threshold)	4 h	During exposure	No differences
Hillert et al. 2007 [50]	PET	12/12	Vanillin, odorant acetone, cedar oil, lavender oil, eugenol, butanol, human pheromones (above odor threshold)	15 s	During task	Activated odor-processing brain regions with odorant-related increase in activation of the anterior cingulate cortex and cuneus–precuneus
Joffres et al. 2005 [51]	SCA, HR, EMG, RR, cognitive test	10/7	Glue, body wash solution, dryer sheet, unscented shampoo	5 min	During task	Increased skin conductance, suggesting involvement of the premotor cortex, hypothalamus, and limbic systems
Kimata 2004 [52]	Plasma SP, VIP, NGF, and histamine, and skin prick tests	25/25	Plastic-based paint with unpleasant odor containing organic solvents	15 min	Before and after exposure	Increased plasma levels of all parameters, suggesting enhanced neurogenic inflammation
Millqvist et al. 2005 [53]	NGF, nasal lavage fluid	13 sensory hyperreactivity /14	Capsaicin	Over 6 min (until inducing coughing)	Before and after exposure	Increased NGF
Orriols et al. 2009 [54]	SPECT	8/8	Plastic-based paint, perfume, petrol, glutaraldehyde (above odor threshold)	3–35 min (until inducing symptoms)	After 15–30 min of exposure	Neurocognitive impairment and dysfunction particularly in odor-processing areas, suggesting a neurogenic origin
Osterberg et al. 2003 [55]	Neurobehavioral test	10/20	n-Butyl acetate, toluene (above odor threshold)	70 min	During exposure	Lower psychological test performance during exposure
Papo et al. 2006 [56]	EEG	23/23	Phenyl ethyl alcohol, hydrogen sulfide (above odor threshold)	200 ms repetition	During task	No differences

Abbreviations: *CI* chemical intolerance, *CO*_*2*_ carbon dioxide, *EEG* electroencephalograph, *EMG* surface electromyogram, *EOG* electrooculogram, *FDG F*-2-fluoro-2-deoxy-D-glucose, *fMRI* functional magnetic resonance imaging, *fNIRS* functional near-infrared spectroscopy, *HR* heart rate, *MCS* multiple chemical sensitivity, *NGF* nerve growth factor, *PET* positron emission tomography, *RR* respiratory rate, *SCA* skin conductance activity, *SP* substance P, *SPECT* single photon-emission computed tomography, *VIP* vasoactive intestinal peptide, *VOC* volatile organic compound

### Exposure event and human response to external stressors

Before describing the results of our review of neurological responses, we describe the primary characteristics of CI and the principal concept on human responses to external environmental factors when considering CI status.

#### Exposure episodes in the onset of CI

CI occurs when individuals are first sensitized via an initial exposure to a certain amount of chemical or repeated exposure to small amounts of chemical (i.e., documentable exposure). On re-exposure, individuals become increasingly sensitized; often, the effects spread, and individuals become sensitized to several additional chemicals [4]. In clinical practice, patients with CI or MCS often report some kind of exposure event that leads to an onset of CI or MCS. Patients with MCS diagnosed in our outpatient department (officially, Outpatient Department of Sick House Syndrome, Hyakumanben Clinic, Kyoto, Japan) experienced episodes of initial exposure to chemicals that first triggered symptoms. For example, they reported exposure to organic solvents, use of pesticides or incense in the workplace, odors from pesticides or exhaust from nearby diesel machines, fragrance from a neighbor, evaporated pesticides used indoors, or chemical exposure after renovation of a house or moving into a newly built home. Other patients had episodes of repeated exposure to solvents emitted from a neighboring industrial plant or paint store, or exposure to fragrances, pesticides, or tobacco smoke emitted in their neighborhood. Patients with MCS subsequently reported a chemical sensitivity condition [32–34]. Other studies also reported initial episodes such as moving into a newly built home, exposure to chemicals at the workplace, use of solvents or pesticides, new carpet, building materials in remodeling, or medications [2, 57–60]. However, there are some cases in which onset was reported as gradual and no specific event or exposure could be recalled by patients [2, 57].

Qualitative or quantitative data on past chemical exposure, and initiating events in particular, are often limited. In particular, quantitative data are almost never reported, and exposure concentrations from transient past events cannot be measured. In one patient, we measured average outdoor air concentrations over 24 h in the house of an MCS patient exposed to solvents emitted from a neighboring industrial plant. Although the patient sometimes suffered due to odor from the plants, we could not detect the solvents at concentrations that exceeded air quality standards or odor thresholds. The results might be affected by diurnal variations or shifting winds. However, the most important feature is that nearly instantaneous elevations (e.g., on the timescale of seconds) of air concentrations, which may exceed odor thresholds, cannot be measured as quantitative air concentrations using existing techniques, even though an individual may respond to the instantaneous exposure to odor and suffer as a result. This leads to the assumption that CI occurs when individuals are sensitized via repeated exposure to small amounts of chemicals, but these phenomena cannot be explained by existing toxicological knowledge. This also calls into question the definition of CI, and evidence does not seem to support the requirement of a specific precipitating exposure event. Thus, specific exposure episodes in individuals with CI and specific modes of action at the onset of CI cannot be adequately examined using existing techniques or risk assessments.

#### Human response to external environmental factors

Humans respond to changes in their external environment in order to maintain homeostasis of their internal environment. Modern human diseases, including obesity, type-2 diabetes, atherosclerosis, autoimmunity, allergy, and certain psychiatric disorders, have two features in common: they involve disruption of homeostasis and are associated with chronic inflammation [61]. The nervous, endocrine, and immune systems play important roles in maintaining homeostasis. Changes in the external environment that affect an organism include exposure to physical, biological, and chemical stresses. The nervous system enacts the initial response against these changes, and the perceived signals are transmitted to the CNS by sensory nerves and are then quickly regulated by autonomic nerves [62].

**Fig. 1:**
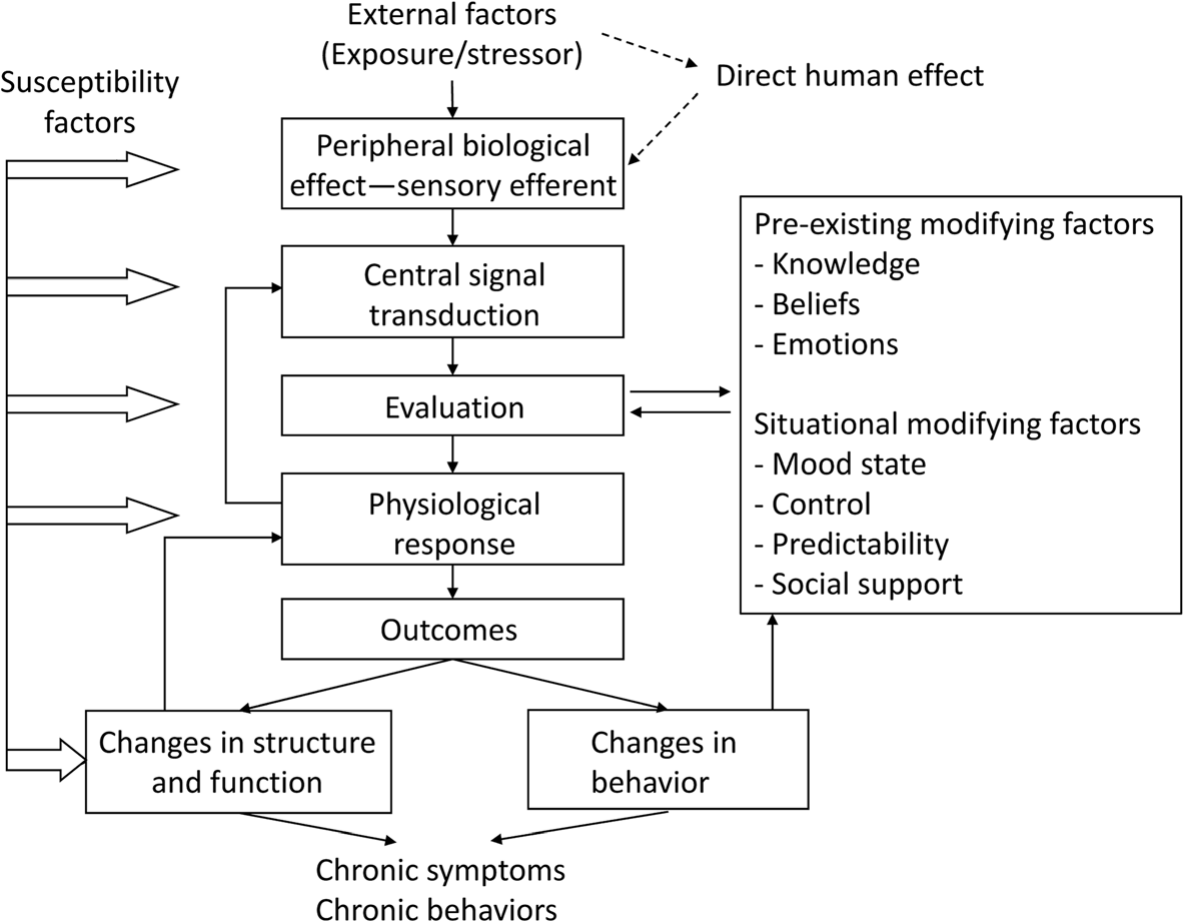
Mechanistic model of the interrelationships among external factors, susceptibility factors, and symptoms (reproduced from Kipen and Fiedler [63] published in *Environmental Health Perspectives* with permission from the authors)

The interrelationship between external stress and CNS responses is often described using a mechanistic model, shown in Fig. 1, of the exposure–outcome relationship of the human stress response [63]. In this model, it is necessary to focus on identifying important susceptibility factors and modifying factors, as well as ultimate changes in behavior, structure, and function in humans. Identifications of these factors, and appropriately and effectively controlling these factors, can result in the prevention of diseases caused by external environmental factors. When investigating the mechanism of action, exposure to external factors and outcomes should be elucidated, thereby defining condition-specific outcomes and triggers. It is also necessary to (i) investigate the loci of the brain involved in processing peripheral nervous system signals from the external environment into the CNS and to (ii) probe the relationship between this processing and modifying factors [63].

MCS appears to involve the sensation of extrinsic substances perceived as hazardous, particularly through olfaction, and involves high sensitivity to various odorous substances [32, 64]. Odor is composed of a single substance, or mixtures of multiple substances, and is cognized through multiple olfactory receptors. Thus, it is important to investigate the processing of these sensations through olfactory perception in the brain. Numerous modes of action have been suggested to explain CI, with the most commonly discussed theories involving the immune, central nervous, olfactory, and respiratory systems, as well as altered metabolic capacity, behavioral conditioning, and emotional regulation [1, 65].

### Neurological responses to chemical exposure

Table 1 summarizes the experimental human studies directly associated with CI or MCS, including IEI due to chemical exposure and related neurological responses or brain imaging studies after exposure to odorous or pungent substances.

Olfactory input directly connects from the olfactory bulb and primary olfactory (piriform) cortex to the amygdala and hippocampus. From these areas, sensory information is conveyed to the secondary olfactory cortices, composed of the orbitofrontal cortex and insular cortex [66]. The olfactory and trigeminal systems are intimately connected and work closely together in the perception of an odorant as most odorants stimulate both sensory systems and despite the fact that trigeminal perception is independent from olfactory processing [67, 68]. A stimulus that activates the trigeminal system evokes cerebral activation of somatosensory regions as well as primary olfactory areas, such as the piriform cortex and orbitofrontal cortex [69].

On the basis of these insights, several studies have used brain imaging to compare metabolic patterns of different brain areas in individuals with CI or MCS versus healthy controls when exposed to odorous or pungent substances. Among the studies shown in Table 1, those assessing cerebral activity in response to several different odorous or pungent stimuli using positron emission tomography (PET), functional magnetic resonance imaging (fMRI), functional near-infrared spectroscopy (fNIRS), and single photon-emission computed tomography (SPECT) found that patients with MCS or CI processed odors differently from controls [32–34, 38, 40, 41, 43, 50, 54].

Hillert et al. reported that regions of the brain engaged in odor processing (the amygdala, piriform cortex, and insular cortex) were less active in MCS patients than in controls in PET imaging when exposed to odorant above the odor threshold. Furthermore, an odorant-related increase in activation of the anterior cingulate cortex (ACC) and cuneus/precuneus was observed [50]. Using fNIRS, Azuma et al. reported increased activation in the prefrontal cortex (PFC) during olfactory stimulation and in the orbitofrontal cortex after olfactory stimulation above the odor threshold in patients with MCS [32–34]. In these studies, subjects were exposed to an odorant for a short period of time (10 or 15 s), and imaging data were collected during and immediately after exposure. Thus, the results reflect instantaneous responses of odor-processing neuronal circuits. Andersson et al. also reported greater reactions in the olfactory region of the orbitofrontal cortex during a 30-s exposure using fMRI, especially in the first 15 s of exposure [40].

Chiaravalloti et al. used brain glucose consumption in PET imaging to show that cortical odor processing in subjects with MCS was characterized by a decrease in activity of the frontal cortex and by activation of the left inferior temporal gyrus [43]. The same group reported a peculiar subcortical activation pattern in subjects with MCS, with enhanced glucose consumption in the bilateral olfactory region, suggesting a potentially useful metabolic index correlated with MCS complaints [38]. These results suggest hyperreactivity and limbic sensitization with neurogenic inflammation. Orriols et al. reported neurocognitive impairment based on SPECT imaging in subjects with MCS and identified brain dysfunction, particularly in odor-processing areas such as the hippocampus, amygdala, and thalamus, when subjects were exposed to odorants above the odor threshold, thereby suggesting a neurogenic origin of MCS [54]. In these studies, subjects were exposed to an odorant for longer durations (9 min and 3–35 min), and imaging data were collected at 24 min or 15–30 min after exposure (compared with previously mentioned studies on the timescale of seconds). Thus, these longer timescale results might reflect affective stress responses after chemical exposure.

Odor processing is instantaneous. An odor can almost instantly key our memory to recall personal events and situations [70, 71]. The function of odor perception is to continually monitor the environment and categorize odors into one of two large categories: those to approach or those to avoid [72]. As shown in Table 1, after the beginning of exposure to odorous or pungent substances, studies reported altered autonomic responses [23, 51], attention bias [39], neurocognitive worsening [54], lower psychological test performance [55], and enhanced neurogenic inflammation [52, 53] in subjects with MCI or CI. However, levels of serum prolactin and cortisol involved in psychological stress [42, 48], oxylipins and endocannabinoids involved in signaling during inflammation [45], cytokines and chemokines in epithelial lining fluid [46], gene expression of inflammatory markers [47], respiratory rate [49], and static electrical parameter [44, 56] were not different compared with healthy controls after exposure to odorous or pungent substances.

### Discussion and outlook

The dorsal portion of the ACC is connected to the PFC and plays an important role in processing top–down and bottom–up stimuli and assigning appropriate control to other areas of the brain [73]. Both the ACC and orbitofrontal cortex are implicated in decision-making, emotion, and social behavior. The orbitofrontal cortex is involved in cognitive processing of stimuli and representation of preferences [74]. CI occurs when individuals are first sensitized via an initial exposure to a certain amount of chemical or repeated exposure to small amounts of chemical. On re-exposure, individuals become increasingly sensitized; often, the effects spread, and individuals become sensitized to several additional chemicals [4]. Numerous studies have reported that odor detection thresholds [34, 50, 64, 75–77] and odor identification [75, 78, 79] occurred at similar levels between subjects with MCS or CI and controls. Although significant differences in odor detection and recognition thresholds were not observed, brain responses at the recognition threshold level were stronger in subjects with MCS [34, 50], and perceived intensity and unpleasantness of odors were significantly higher for subjects with MCS [80].

Human episodic memory involves the long-term memory process that enables one to mentally and consciously relive specific personal events from the past [81, 82]. In particular, episodic odor memory undergoes extremely little long-term loss compared with memories of pictures or odor presented in a laboratory environment [83], and odors appear to trigger the most vivid and emotional memories [84]. Emotional processing related to stimulation with or discomfort to odor prevails, and the consequences of the processing appear rapidly [66, 83]. The PFC regulates the formation and control of memory [85, 86] and plays an important role in long-term odor memory [71, 87].

Individuals with CI exhibit stronger physical and psychological reactions to odorous or pungent substances at normally perceived levels in daily life than healthy people. This status persists due to repeated daily exposure to these substances, and they exhibit physical and psychological intolerance to the substances at levels less than those established to have harmful effects in the general population [32]. Thus, in the mechanism of CI, past exposure is stored as memory in the PFC connected from the ACC through olfactory nerve circuits. The processing of top–down stimuli from these cortices involves the central system related to emotional and the autonomic nervous system, and various physical or psychological symptoms may be induced in subjects with CI in later life when individuals with CI are exposed to odorous or pungent substances. Such responses in odor processing are associated with cognition and memory processes in the brain and occur when an individual distinguishes between a nonagent substance and an agent substance (i.e., approach or avoid). This means that the mode of action in this response is not specific to individual substances, but is associated with a past event such as exposure to odorous or pungent substances, and this mode of action involves responses to multiple chemicals in CI. Interestingly, it has been reported that symptoms can occur with odors associated with negative events [88] and generalization of acquired somatic symptoms in response to odorous substances [89].

Although some studies reported neurogenic inflammation after exposure to odorous or pungent substances, including hypoactivation of frontal and prefrontal areas [38, 43], these results might reflect affective stress responses after chemical exposure. Dantoft et al. reported increased levels of proinflammatory cytokines, including interleukin-1β (IL-1β), interleukin-6 (IL-6), and tumor necrosis factor-α (TNF-α), and inverse regulation of Th2-associated cytokines interleukin-4 and interleukin-13, in unexposed subjects with MCS, suggesting a deviating Th2-associated cytokine response not involving IgE-mediated mechanisms [90]. A similar profile of proinflammatory mediators, including increased levels of IL-1β, IL-6, and TNF-α, has been reported in depression and stress, which are thought to be inflammatory responses of microglial activation after stress or environmental cues such as stranger and danger signals [91–94]. Studies on the involvement of brain microglia in stress have been reported since 2005 [95], and the association with acute stress was first reported in 2007 [96]. Exploring the involvement of microglia in CI may provide new insight into the mechanisms of CI. In addition, studies have shown an association of increased severity of post-traumatic stress disorder (PTSD) and neuropsychological performance with decreased medial PFC and rostral ACC activity [97], as well as an association of greater severity of depression and somatic symptoms with less synaptic density in the dorsolateral PFC, ACC, and hippocampus [98]. Thus, neurogenic inflammation in CI or MCS is considered to be the brain’s response to stress or negative psychosomatic status after exposure to unpleasant substances rather than a result of toxicological effects.

**Fig. 2:**
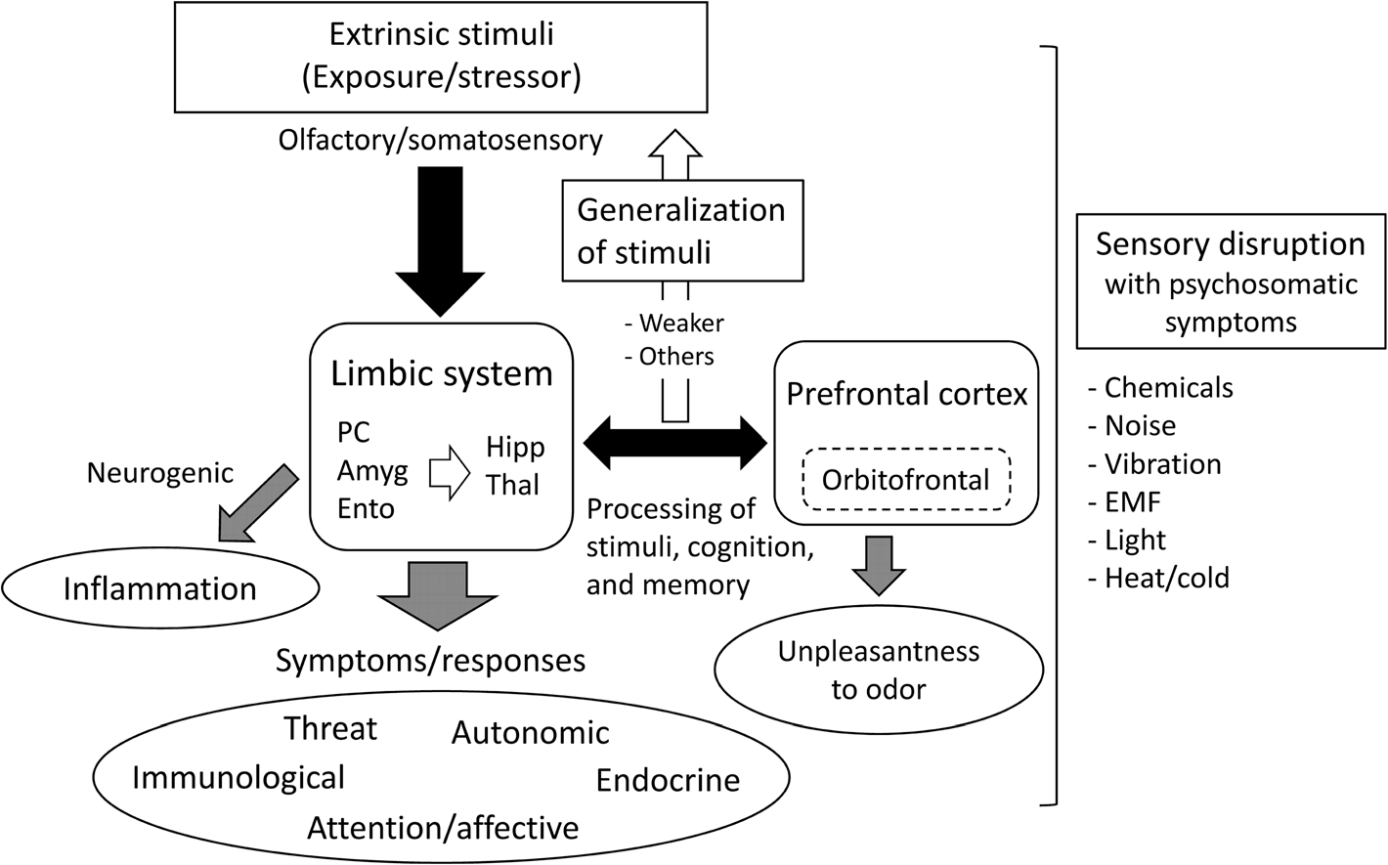
Sensory and cognition model of the interrelationships among stimulus factors, limbic system, cortices, symptoms, and responses. Abbreviations: Amyg, amygdala; EMF, electromagnetic field; Ento, entorhinal cortex; Hipp, hippocampus; PC, piriform cortex; Thal, thalamus

On the basis of these studies, we suggest a sensory and cognition model of the interrelationships between extrinsic stimuli, the limbic system, cortices, symptoms, and responses (Fig. 2). Nordin proposed central sensitization as a possible mechanism of CI and called this a chemosensory model [99]. The results of our review and this model led to a great outlook for derivation of our model. We proposed a sensory and cognition model (Fig. 2) based on the mechanistic model shown in Fig. 1 and also considered the importance of processing extrinsic stimuli and cognition of sensation involving the limbic system and PFC. When individuals first receive strong stimulation or stress via an initial exposure to a certain amount of chemical or repeated exposure to small amounts of chemical, the stimulation or stress is strongly cognized by an odor-processing neuronal circuit and stored as memory in the PFC area through the limbic system; the stimulus is subsequently generalized. Once generalized, even if individuals are later exposed to weaker stimuli, they recognize it as strong stimulation or stress, which induces various symptoms through the limbic system and PFC. In light of this model, distracting attention from odorous or pungent substances may be effective for the recovery from CI status, resulting in the treatment of CI. In the clinical practice, adequate assessment of exposure history for identifying the agent substance which should be avoided would have an important role for the recovery of a patient with CI [100]. Interestingly, a 5-year follow-up study reported that appropriate physical activity and maintaining a regular lifestyle, including diet or sleep, were significant factors for the improvement of CI [101]. However, the detailed neurological and pathological mechanism through the limbic system and the role of PFC or orbitofrontal cortex in the onset of CI and appearance of symptoms in individuals with CI remain unclear. Similarly increased PFC activity is suggested in incidences of experimental pain under chronic clinical pain conditions [102]. Further research into these mechanisms is required.

Three 5-year follow-up studies reported that participants who developed CI between baseline and follow-up reported more health complaints or negative psychosomatic states at baseline compared to participants who did not develop CI [101, 103, 104]. An association of risk of acquiring CI with measures of inherent physical constitution, such as cold sensitivity or the presence of an indoor cat during childhood, has also been reported [105]. In this study, the prevalence of current CI in mothers was 4.5% and in their 3-year-old children was 0.25%, or approximately one eighteenth of that reported by their mothers [105]. Devriese et al. reported that generalization of acquired somatic symptoms in response to odorous substances especially occurred in individuals with high negative affectivity [89]. Thus, negative status or inherent constitution may cause greater susceptibility to CI. In addition, one’s housing environment in childhood is an important risk factor for CI. Finally, such a generalized pathological condition due to disturbance of sensation may lead to the symptoms induced by other stressors such as noise, vibration, electromagnetic field, light, or excess heat or cold. Such conditions can be regarded as a sensory disruption with psychosomatic symptoms (Fig. 2).

## Conclusions

This review highlights evidence from studies conducted during the past two decades on our understanding of brain function and networks after exposure to extrinsic stimuli and how these relate to CI status. As the review indicates, our understanding of the mechanisms of CI has gradually increased by using chemical provocation tests along with brain imaging techniques, and these studies have made multiple contributions to elucidate the mode of CI. There is consistent evidence that altered neurological processing of sensory information contributes to CI status. However, neurophysiological research exploring the processing of extrinsic stimuli and cognition of sensation through the limbic system and related cortices in the onset of CI is required. Future research elucidating the mechanisms of CI will impact clinical practice and thus contribute to a decreased prevalence of CI in society.

## Data Availability

Not applicable.

## Abbreviations

ACC: Anterior cingulate cortex
Amyg: Amygdala
CI: Chemical intolerance
CNS: Central nervous system
CO_2_: Carbon dioxide
EEG: Electroencephalograph
EMF: Electromagnetic field
EMG: Surface electromyogram
Ento: Entorhinal cortex
EOG: Electrooculogram
FDG: *F*-2-fluoro-2-deoxy-D-glucose
fMRI: Functional magnetic resonance imaging
fNIRS: Functional near-infrared spectroscopy
Hipp: Hippocampus
HR: Heart rate
IEI: Idiopathic environmental intolerance
IL-1β: Interleukin-1β
IL-6: Interleukin-6
MCS: Multiple chemical sensitivity
NGF: Nerve growth factor
PC: Piriform cortex
PET: Positron emission tomography
PFC: Prefrontal cortex
PTSD: Post-traumatic stress disorder
RR: Respiratory rate
SCA: Skin conductance activity
SP: Substance P
SPECT: Single photon-emission computed tomography
Thal: Thalamus
TNF-α: Tumor necrosis factor-α
VIP: Vasoactive intestinal peptide
VOC: Volatile organic compound
